# TBX2 over-expression promotes nasopharyngeal cancer cell proliferation and invasion

**DOI:** 10.18632/oncotarget.17084

**Published:** 2017-04-13

**Authors:** Yan Lv, Meng Si, Nannan Chen, Ya Li, Xingkai Ma, Huijun Yang, Ling Zhang, Hongyan Zhu, Guang-yin Xu, Ge-ping Wu, C. Cao

**Affiliations:** ^1^ Center of Translational Medicine, The First People Hospital of Zhangjiagang City, Soochow University, Suzhou, China; ^2^ Department of Neurology, The Second Affiliated Hospital of Soochow University, Suzhou, China; ^3^ Institute of Neuroscience, Soochow University, Suzhou, China; ^4^ Department of Otolaryngology, The First People Hospital of Zhangjiagang City, Soochow University, Suzhou, China

**Keywords:** TBX2, nasopharyngeal cancer, invasion, proliferation, siRNA

## Abstract

TBX2 is a member of the T box transcription factor family. Its expression and potential biological functions in nasopharyngeal cancer (NPC) cells are studied here. We showed that TBX2 mRNA and protein expression was significantly elevated in multiple human NPC tissues, as compared with that in adjacent normal tissues. Knockdown of TBX2 by targeted-siRNA significantly inhibited proliferation and invasion of NPC cells (CNE-1 and HONE-1 lines). Meanwhile, TBX2 knockdown also induced G1-phase cell cycle arrest. At the molecular level, we discovered that expressions of several tumor suppressor genes, including *p21, p27*, *phosphatase with tensin homology (PTEN)* and *E-Cadherin*, were increased dramatically after TBX2 knockdown in above NPC cells. Collectively, our results imply that TBX2 over-expression promotes NPC cell proliferation and invasion, possibly via silencing several key tumor suppressor genes.

## INTRODUCTION

Nasopharyngeal cancer (NPC) is a rare type of head and neck squamous cell carcinoma (HNSCC). NPC could be caused by a combination of factors, including Epstein-Barr virus infection, environmental influences, and heredity [1–3]. NPC is endemic in Southeast Asia and southern China. In 2008, over 84,400 cases of NPC were diagnosed, and 80% of the cases occurred in Asia [4]. Even with the development of modern radiation therapy, distant metastasis will develop in 30-40% of patients within 4 years [5]. Improved understanding of NPC progression and the development of novel therapies is still urgently needed.

TBX2 is a member of the T box transcription factor family proteins, which are characterized by a highly conserved T box DNA binding domain [6]. TBX2 is fundamental to the morphogenesis of various tissues and organs, such as limbs [7, 8], heart [9], bone [10, 11] and mammary glands [12]. Existing evidences have linked TBX2 to tumorigenesis. TBX2 expression is up-regulated in a wide range of cancers, including melanoma [13], pancreatic cancer [14], bladder cancer, breast cancer [15–17], colorectal cancer [18] and lung cancer [19].

TBX2 functions as a repressor of transcription, which inhibits promoter activity of a number of cancer-suppressing genes. For example, it can facilitate the bypass of senescence by repressing transcription of *Cdkn2a (p19ARF)* and *Cdkn1a (p21WAF1)* [17]. TBX2 could also promote anchorage-independence growth and increase resistance to apoptotic stimuli in p53-negative SW13 adrenocortical carcinoma cells [20]. Furthermore, TBX2 is shown to directly repress E-Cadherin transcription, and to increase motility and invasiveness of malignant breast cancer cells [21]. Meanwhile, TBX2 directly targets checkpoint proteins p21 and p27 to promote cell cycle progression [21, 22]. Despite the importance of TBX2 in tumorigenesis of various cancers, the expression and biological functions of TBX2 in NPC are still largely unknown. In the current study, we report that TBX2 expression is up-regulated in clinical NPC tissue samples. Knockdown of TBX2 in the NPC cells inhibits cancer cell proliferation and invasion.

## RESULTS

### TBX2 expression in human NPC tissues

**Figure 1:**
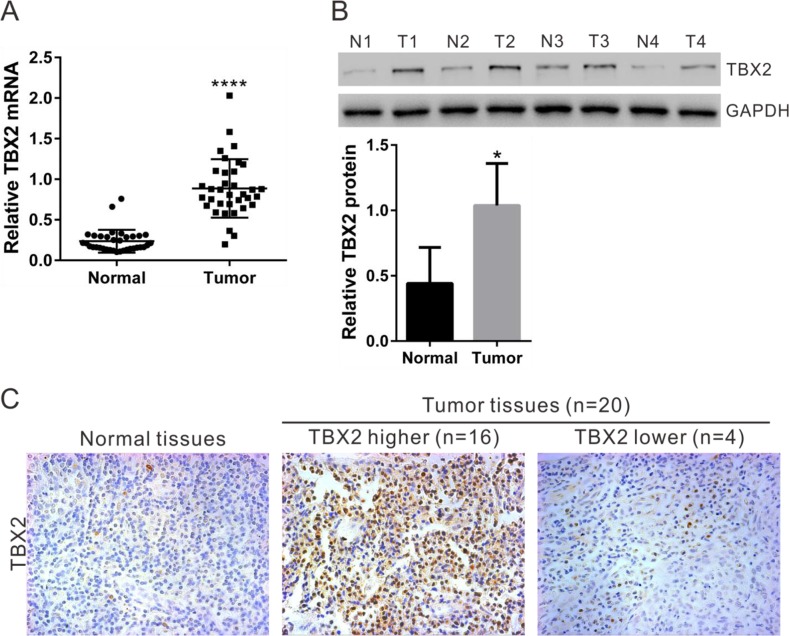
TBX2 expression in human NPC tissues **(A)** The mRNA expression of *TBX2* in NPC (“Tumor”) and paired surrounding normal tissues (“Normal”, n=35) were examined by qRT-PCR. **(B)** Representative TBX2 protein expression in NPC (T1, T2, T3 and T4) and normal tissues (N1, N2, N3 ad N4). GAPDH was used as a loading control. **(C)** Immunohistochemistry staining of TBX2 in NPC and normal tissues. **** *P* < 0.0001 vs. “Normal” tissues **(A)**.* *P* < 0.01 vs. “Normal” tissues **(B)**.

To investigate the expression of TBX2 in NPC, TBX2 mRNA expression was assessed in RNA samples that were isolated from 35 pairs of NPC tumor tissues and paired adjacent normal tissues. Quantitative real-time PCR assay (qRT-PCR) results in Figure 1A showed that the average TBX2 mRNA value in NPC tissues was significantly (*P*<0.0001) higher than in normal samples. TBX2 protein expression was also higher in NPC tissues, which was determined by Western blot assay (Figure 1B) and immunohistochemistry (IHC) staining assay (Figure 1C). For the Western blot assay, blot images analyzing four representative paired-tissues were presented (Figure 1B, upper panel). All the blot data of 35 paired-tissues were integrated for statistics analysis (Figure 1B, lower panel). Twenty of the 35 paired-tissues showed fine IHC staining (Figure 1C). Sixteen of them (80%) demonstrated high TBX2 expression, and representative images were shown (Figure 1C). These data suggest that TBX2 protein and mRNA expression is significantly increased in NPC tissues.

### siRNA knockdown of TBX2 in NPC cells

**Figure 2:**
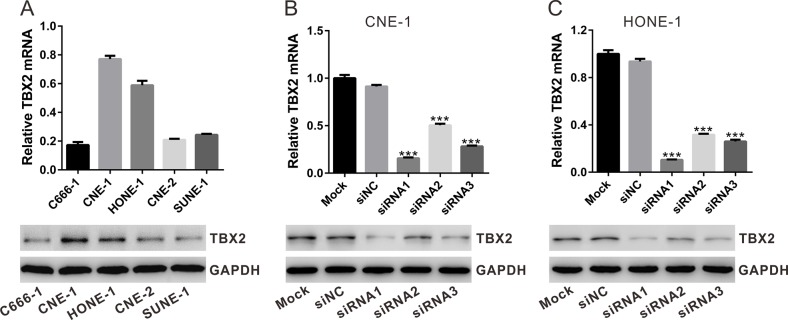
siRNA knockdown of TBX2 in NPC cells **(A)** mRNA (upper panel) and protein (lower panel) levels of TBX2 in five listed NPC cell lines. **(B, C)** Identification of TBX2 knockdown efficiency by qRT-PCR and Western blotting in CNE-1 cells and HONE-1 cells. All data are representative results of three independent experiments. Mock: non-treated cells; siNC: cells transfected with siNC; siRNA1-3: cells transfected with TBX2 siRNA1, siRNA2 or siRNA3. ***P<0.001 vs. siNC.

We also tested expression of TBX2 in a panel of NPC cell lines, including C666-1, CNE-1, HONE-1, CNE-2 and SUNE-1. As shown in Figure 2A, among all the tested cell lines, CNE-1 and HONE-1 showed highest expression of TBX2 (both protein and mRNA). In order to study the function of TBX2 in NPC cell behaviors, we transfected TBX2 siRNAs (siRNA1, siRNA2 and siRNA3, with non-overlapping sequence) or control siRNA (siNC) into CNE-1 and HONE-1 NPC cells. To determine the effect of siRNA on TBX2 expression, qRT-PCR and Western blotting were performed. Relative to “Mock” or siNC-transfected cells, transfection of the TBX2 siRNAs in CNE-1 (Figure 2B) and HONE-1 (Figure 2C) cells significantly decreased TBX2 mRNA and protein expression. Among all the tested siRNAs, TBX2 siRNA1 had the highest knockdown efficiency, and was thus chosen for further experiments.

### TBX2 knockdown inhibits NPC cell proliferation and cell-cycle progression

**Figure 3:**
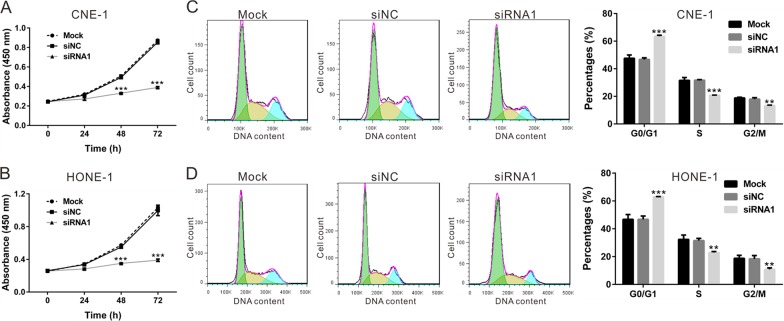
TBX2 knockdown inhibits NPC cell proliferation and cell-cycle progression CNE-1 cells and HONE-1 cells were transfected with siNC or TBX2 siRNA1. **(A, B)** Cell viability was determined at indicated time points by CCK-8 assay. **(C, D)** At 48h post transfection, cell cycle distribution analysis was performed after PI staining. All assays were performed in triplicate. All data are representative results of three independent experiments. Mock: non-treated cells; siNC: cells transfected with siNC; siRNA1: cells transfected with TBX2 siRNA1. **P<0.01, ***P<0.001 vs. siNC.

The CCK-8 assay was applied to determine the function of TBX2-siRNA1 on the proliferation of CNE-1 (Figure 3A) and HONE-1 NPC cells (Figure 3B). As demonstrated, TBX2 siRNA1 transfection indeed significantly inhibited above NPC cell proliferation at 48h and 72h (Figure 3A and 3B). Accumulating evidence has suggested a function of TBX2 in promoting cell cycle progression [23]. We then examined whether TBX2 siRNA1 could also inhibit cell cycle progression. Propidium iodide (PI) staining of DNA content was applied to analyze cell cycle progression, and results showed that the G0/G1 phase percentage in TBX2 siRNA1-transfected CNE-1 cells (Figure 3C) and HONE-1 cells (Figure 3D) was 63.47 ± 0.80% and 62.95 ± 0.22%, as compared to 46.69 ± 1.24% and 46.80 ± 2.35 % in siNC cells. Concurrently, S-phase and G2/M-phase cells were decreased in NPC cells transfected with TBX2 siRNA1 (Figure 3C and 3D). These results show that TBX2 knockdown inhibits NPC cell proliferation and cell-cycle progression.

### Silencing of TBX2 inhibits NPC cell *in vitro* migration

**Figure 4:**
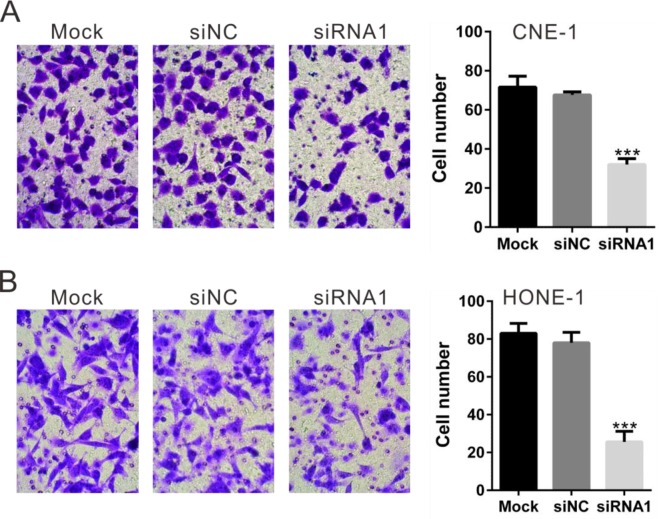
Silencing of TBX2 inhibits NPC cell *in vitro* migration Invasion assay was carried out in Matrigel-coated Transwell chambers. Cells that migrated into the lower well were stained, photographed and counted. TBX2 silencing in CNE-1 cells **(A)** and HONE-1 **(B)** cells notably inhibited cell invasion. All data are representative results of three independent experiments. Mock: non-treated cells; siNC: cells transfected with siNC; siRNA1: cells transfected with TBX2 siRNA1. ***P<0.001 vs. siNC.

In order to study whether TBX2 siRNA could change the invasion ability of CNE-1 cells and HONE-1 cells, we transfected TBX2 siRNA1 again in these cell lines. *In vitro* invasion assay was performed in Matrigel-pre-coated Transwell. Results showed that, as compared to cells transfected with siNC, the invasive potential of CNE-1 cells and HONE-1 cells with TBX2 siRNA1 was dramatically inhibited (Figure 4A and 4B). The number of migrated cells decreased by about 52% (for CNE-1 cells) and 68% (for HONE-1 cells) following TBX2 siRNA knockdown (Figure 4A and 4B). Therefore, TBX2 knockdown also inhibits NPC cell *in vitro* migration. Notably, for the migration assay, mitomycin C (5 μg/mL) was always added to exclude the possible influence of cell proliferation. As shown in Supplementary Figure 1, treatment with mitomycin C (5 μg/mL) failed to decrease CCK-8 viability OD of CNE-1 cells (Supplementary Figure 1A) and HONE-1 cells (Supplementary Figure 1B). Further, CNE-1 and HONE-1 cell proliferation was indeed blocked with mitomycin C treatment, as the viable cell number was almost unchanged after culture for 12-48 hours (Supplementary Figure 1C and 1D).

### TBX2 siRNA knockdown increases expression of several key tumor-suppressors

**Figure 5:**
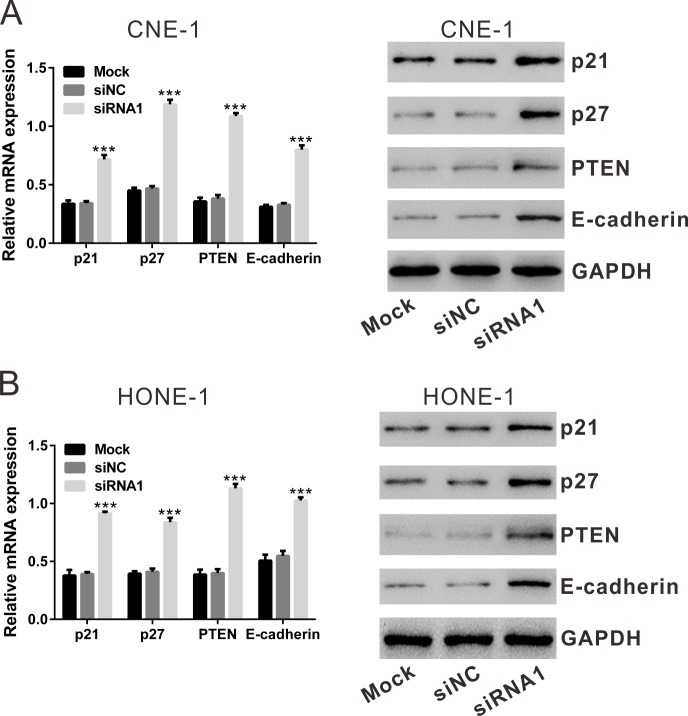
Effect of TBX2 siRNA1 on the mRNA and protein expression of p21, p27, PTEN and E-Cadherin by qRT-PCR and Western blotting assay, respectively **(A)**, CNE-1 cells. **(B)**, HONE-1 cells. Mock: non-treated cells; siNC: cells transfected with siNC; siRNA1: cells transfected with TBX2 siRNA1. ***P<0.001 vs. siNC.

The above results demonstrated that TBX2 siRNA knockdown inhibits proliferation and migration of human NPC cells. Previous studies have suggested that TBX2 can repress tumor-suppressors phosphatase with tensin homology (PTEN) [24], p21 [25, 26], p27 [26] and E-Cadherin [21], thus promoting cell proliferation and invasion. We then tested the effect of TBX2 siRNA knockdown on the expression of the above genes. The qRT-PCR data showed that silencing TBX2 remarkably increased the mRNA expression of *PTEN* (increase by: CNE-1 cells, 184.8% and HONE-1 cells, 183.4%), *p21* (increase by: CNE-1 cells, 109.5% and HONE-1 cells, 133.9%), *p27* (increase by: CNE-1 cells, 153.7% and HONE-1 cells, 104.0%) and *E-Cadherin* (increase by: CNE-1 cells,143.4% and HONE-1 cells, 87.1%) (Figure 5A and 5B, left panels). Similar changes were obtained at protein levels, PTEN, p21, p27 and E-Cadherin protein expression was significantly increased in TBX2-silenced cells (Figure 5A and 5B, right panels). As expected, siNC showed no effect on protein and mRNA expression of above genes. These data together suggest that TBX2 siRNA knockdown increases expressions of several key tumor-suppressors, which could be responsible for NPC cell proliferation and migration inhibition.

## DISCUSSION

Up-regulation of TBX2 has been reported in a wide range types of cancer [13–19]. In the present study, we reported that TBX2 mRNA and protein expression was significantly higher in NPC tissues samples than that in the adjacent normal tissues. Previous studies have implied a possible function of TBX2 up-regulation in promoting cell proliferation and invasion. Here, we also observed similar results in NPC cells, indicating that TBX2 might also promote proliferation and invasion of NPC cells.

Uncontrolled cellular proliferation is one of the main features of cancer. Here, siRNA knockdown of TBX2 in two NPC cell lines significantly decreased cancer cell proliferation, which was consistent with previous studies in heart cells [27], in melanoma cells [13] and rhabdomyosarcoma cells [28]. The checkpoint protein p21 is a well-known cyclin-Cdk inhibitor, which is vital for the G1-S progression [29]. Over-expression of p21 shall cause G1-S arrest [29]. Similarly, p27 is a potent inhibitor of cyclin D1-Cdk4 and cyclin A-Cdk2 protein kinase activity, which is related to p21 [22]. Exogenous expression of p27 is shown to induce G1-S arrest as well [22]. Both p21 [25, 26], p27 [26] are the TBX2-targted proteins. In the current study, we showed that p21 and p27 were increased in TBX2-silenced NPC cells, that could explain the subsequent G1 arrest and proliferation inhibition.

PI3K/AKT pathway is involved in the regulation of cell cycle progression and cellular growth [30]. Recent work has shown that TBX2 is a central component of the PTEN/PI3K/AKT signaling pathway in rhabdomyosarcoma cells by repressing PTEN [24]. Consistent with these findings, we showed that PTEN expression was increased in TBX2-silenced cells. Therefore, TBX2 may exert the cell proliferation-promoting functions via regulating PTEN/PI3K/AKT signaling pathway as well.

Cell invasion/migration is essential to the metastasis of cancer. Increased expression of E-Cadherin was shown to inhibit cell invasion [31]. Here, we discovered that TBX2 knockdown significantly increased E-Cadherin expression, and repressed *in vitro* invasion/migration of NPC cells. Our results here are consistent with the findings in malignant breast cancer cells [21]. These results along with the mentioned previous studies suggest that TBX2 could promote cell proliferation and invasion of different cancer cells. Recent studies have also proposed a pivotal function of TBX2 in cell senescence [17]. It has been shown that TBX2 can inhibit *Cdkn2a* (*p19ARF*) promoter and suppress E2F1, Myc or HRAS-mediated induction of *Cdkn2a* (*p19ARF*) [17]. Therefore, it would be interesting to test potential effect of TBX2 in NPC cell senescence.

## MATERIALS AND METHODS

### Clinical tissue specimens

Paired human NPC tissues and matched normal tissues were collected from 35 written-inform consent patients underwent standard surgical procedures in The First People Hospital of Zhangjiagang City (Zhangjiagang, China). Tissue samples used for RNA isolation or protein extraction were immediately snap-frozen in liquid nitrogen and stored at −80°C. Other tissue samples for histological examination and immunohistochemistry staining were fixed using 10% buffered formalin. The experiment protocols were approved by the Institutional Review Committees of The First People Hospital of Zhangjiagang City and Soochow University.

### RNA isolation and quantitative RT-PCR (qRT-PCR)

Total RNA was isolated from NPC tissues, normal tissues samples and cell lines with TRIzol reagents (Life Technologies), and then reversely transcripted to cDNA with the First-Strand cDNA Synthesis Kit (Thermo Fisher, Shanghai, China) following the manufacturers’ instructions. The quantitative real-time PCR reactions were performed on a 7300 Real Time PCR machine (Applied Biosystems, Foster City, CA) with SYBR Green qPCR Master Mixes (Thermo Fisher). GAPDH gene was amplified as an internal control. The oligonucleotides used as PCR primers were listed in Table 1.

**Table 1:** Primer sequences for quantitative PCR

Primer	Primer sequence	Size (bp)
TBX2 (NM_005994.3)	F: 5′- GACCCTGAGATGCCCAAACG -3′ R: 5′- GGTGCGGAAGGTGCTGTAAG -3′	222
p21 (NM_000389.4)	F: 5′- TAGCAGCGGAACAAGGAG -3′ R: 5′- AAACGGGAACCAGGACAC -3′	249
p27 (NM_004064.4)	F: 5′- GCTTGCCCGAGTTCTACTAC -3′ R: 5′- GCAGGTCGCTTCCTTATTCC -3′	220
PTEN (NM_000314.6)	F: 5′- ATTAGTGCTGTTGCTAGTTC -3′ R: 5′- GCAACAATCATTAGGCTTTC -3′	194
E-Cadherin (NM_004360.3)	F: 5′- GAGAACGCATTGCCACATACAC -3′ R: 5′- AAGAGCACCTTCCATGACAGAC -3′	164
GADPH (NM_001256799.2)	F: 5′- CACCCACTCCTCCACCTTTG -3′ R: 5′- CCACCACCCTGTTGCTGTAG -3′	110

### Protein extraction and western blotting assay

Cells or human tissue specimens were lysed via the described lysis buffer [32–34]. Aliquots of 40 μg of total proteins from each treatment were separated by 10-12% SDS-PAGE gels and were transferred onto polyvinylidene difluoride (PVDF) membranes (Millipore, Bedford, MA), which were then blocked in 10% milk and incubated with specific primary and second antibodies. Antibody-antigen binding was detected with the enhanced chemiluminescence (ECL) detection system (Amersham Biosciences, Piscataway, NJ). Each band was quantified through Image J software (NIH). For the Western blot assay, each lane was loaded with exact same amount of quantified protein lysates (40 μg per sample). Same set of lysate samples were run in sister gels to test different proteins. Following antibodies were utilized: TBX2 (ab33298, Abcam, Cambridge, MA, 1:1000 dilution), PTEN (9552, Cell Signaling; Danvers, MA, 1:1000 dilution), E-Cadherin (3195, Cell Signaling, 1:1000 dilution), p21 (2946T, Cell Signaling, 1:2000 dilution), p27 (3688, Cell Signaling, 1:1000 dilution) and GAPDH (2188, Cell Signaling, 1:1000 dilution). Secondary antibodies (A0208, HRP-labeled Goat Anti-Rabbit IgG, Beyotime, 1:1000 dilution; A0216, HRP-labeled Goat Anti-Mouse IgG, Beyotime, 1:1000 dilution) were employed.

### Immunohistochemistry analysis

TBX2 protein expression in tissue sections was detected by immunohistochemistry (IHC). Briefly, 5-μm sections were de-paraffinized in xylene (concentration over 99.0%), rehydrated through a graded ethanol series, and antigen-retrieved by heat treatment in 10mM citrate buffer for 5 min. To inhibit endogenous peroxidase, sections were treated with 0.3% hydrogen peroxide for 30 min. After incubation with 5% BSA for 30 min to block non-specific binding, sections were incubated with anti-TBX2 antibody (ab33298, Abcam, 1:50 dilution) overnight at 4°C. The sections were then incubated with horseradish peroxidase (HRP)-conjugated secondary antibody (A0208, Anti-Rabbit IgG, Beyotime, 1:1000 dilution) at room temperature for 1h and developed with the DAB kit (Vector Laboratories, Burlingame, CA) followed by counterstained with Mayer's hematoxylin. Section images were obtained using a microscope (Nikon, Japan).

### Cell culture

Human NPC cell lines, C666-1, CNE-1, HONE-1, CNE-2 and SUNE-1, were obtained from Shanghai Institutes for Biological Science Cell Bank (Shanghai, China) and cultured in RPMI 1640 medium (Life Technologies, Shanghai, China) supplemented with 10% fetal bovine serum (FBS, Hyclone, Shanghai, China), 1% penicillin-streptomycin solution in a 37°C incubator containing 5% CO_2_. For the utilized NPC cell lines, DNA fingerprinting and profiling were performed every 4 months to confirm the origin of the cell lines, and to distinguish the cell line from cross-contamination. All cell lines were subjected to mycoplasma and microbial contamination examination. Population doubling time, colony forming efficiency, and morphology under phase contrast were also measured every 6 months under defined conditions to confirm the phonotype of cell line.

### RNA interference

Three TBX2 siRNAs (siRNA1: 5′- CCAAAC GCAUGUACAUCCA-3′, siRNA2: 5′- CCAUCCUA AACUCCAUGCA-3′ and siRNA3: 5′- CACAGCUGA AGAUCGACAA-3′) and a non-specific scramble siRNA (siNC) were synthesized by GenePharm (Shanghai, China), and transfected into CNE-1 cells and HONE-1 cells with Lipofectamine 2000 (Invitrogen, Carlsbad, CA) per the manufacture's instruction. Knockdown efficiency was determined by qRT-PCR and Western blotting at 48h after transfection.

### Cell viability assay

CNE-1 cells and HONE-1 cells were plated onto 96-well plates at a density of 2 × 10^3^ cells per well and transfected with siRNA1 or siNC. After incubation for 0, 24, 48 and 72 h, 10 μL cell counting assay kit-8 (CCK-8) solution (Dojindo Laboratories, Japan) was added to all wells and incubated at 37°C for another 1 h. The absorbance at 450 nm was measured using a microplate reader (Labsystems, Helsinki, Finland).

### Cell cycle distribution analysis

Cells were collected at 48h after siRNA transfection, washed with PBS and fixed in ice-cold ethanol at −20°C overnight. The cells were then washed with PBS, and stained with staining solution containing 20 μg/mL propidium iodide (PI, Sigma, St. Louis, MO) and100 μg/mL RNase (Sigma) for 20 min. DNA content was analyzed with a flow cytometer (BD Biosciences, Franklin Lakes, NJ).

### *In vitro* invasion assays

Invasion assays were performed using chamber with 8 μm pore filters (Corning, New York, NY) pre-coated with 1 mg/mL Matrigel (BD Biosciences, Shanghai, China). Briefly, CNE-1 cells and HONE-1 cells were transfected with siRNA1 or siNC. At 24h post transfection, the cells were incubated in serum-free medium overnight. The cells were collected and exact 5 × 10^4^ cells in serum-free medium were plated to the upper chamber, while medium containing 10% FBS was added to the lower chamber. After 24h of incubation, cells on the lower surface of the membrane were fixed with 4% paraformaldehyde, stained with 0.5% crystal violet and then counted in five random fields. To block cell proliferation, mitomycin C (5 μg/mL, Sigma, Shanghai, China) was added.

### Statistical analysis

Individual culture dishes or wells were analyzed separately (no pooling of samples was used). In each experiment a minimum of three wells/dishes of each treatment was used. Each experiment was repeated a minimum of three times, in each experiment, the mean value of the repetitions was calculated and this value was used in the statistical analysis. The data was presented as mean ± standard deviation (SD). One way ANOVA analysis was used to analyze the significance of *in vitro* experiments. *P* value less than 0.05 was considered statistically significant.

## CONCLUSIONS

In conclusion, our results demonstrates that the expression of TBX2 is markedly overexpressed in NPC tissues. Knockdown of TBX2 significantly inhibits the NPC cell proliferation and invasion, and induces cell cycle arrest.

## SUPPLEMENTARY MATERIALS FIGURE


