# Supplementary material for: TBX2 over-expression promotes nasopharyngeal cancer cell proliferation and invasion
Source: Oncotarget. 2017 Apr 13;8(32):52699–707. doi: 10.18632/oncotarget.17084 (PMC5581062; doi:10.18632/oncotarget.17084)
Supplement: Supplementary file 1 [file oncotarget-08-52699-s001.pdf]

TBX2 over-expression promotes nasopharyngeal cancer cell proliferation and invasion

SUPPLEMENTARY FIGURE

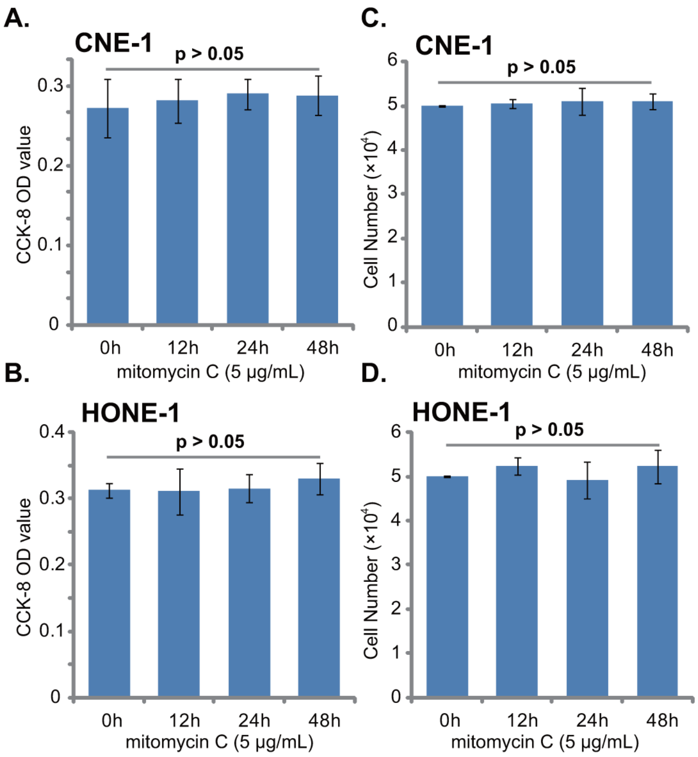

Supplementary Figure 1: CNE-1 cells (A and B) and HONE-1 cells (C and D) were treated with mitomycin C (5 µg/mL) for indicated time; Cell viability was tested by CCK-8 assay (A and C), and viable cell ("Trypan blue negative" cell) number was recorded (B and D). All data are representative results of three independent experiments.
